# Surveillance-ready transcription: nuclear RNA decay as a default fate

**DOI:** 10.1098/rsob.170270

**Published:** 2018-03-21

**Authors:** Stefan Bresson, David Tollervey

**Affiliations:** Wellcome Centre for Cell Biology, University of Edinburgh, Edinburgh, EH9 3BF, UK

**Keywords:** RNA surveillance, gene expression, quality control, RNA processing

## Abstract

Eukaryotic cells synthesize enormous quantities of RNA from diverse classes, most of which are subject to extensive processing. These processes are inherently error-prone, and cells have evolved robust quality control mechanisms to selectively remove aberrant transcripts. These surveillance pathways monitor all aspects of nuclear RNA biogenesis, and in addition remove nonfunctional transcripts arising from spurious transcription and a host of non-protein-coding RNAs (ncRNAs). Surprisingly, this is largely accomplished with only a handful of RNA decay enzymes. It has, therefore, been unclear how these factors efficiently distinguish between functional RNAs and huge numbers of diverse transcripts that must be degraded. Here we describe how bona fide transcripts are specifically protected, particularly by 5′ and 3′ modifications. Conversely, a plethora of factors associated with the nascent transcripts all act to recruit the RNA quality control, surveillance and degradation machinery. We conclude that initiating RNAPII is ‘surveillance ready’, with degradation being a default fate for all transcripts that lack specific protective features. We further postulate that this promiscuity is a key feature that allowed the proliferation of vast numbers of ncRNAs in eukaryotes, including humans.

## 1. Introduction

**Figure 1.**
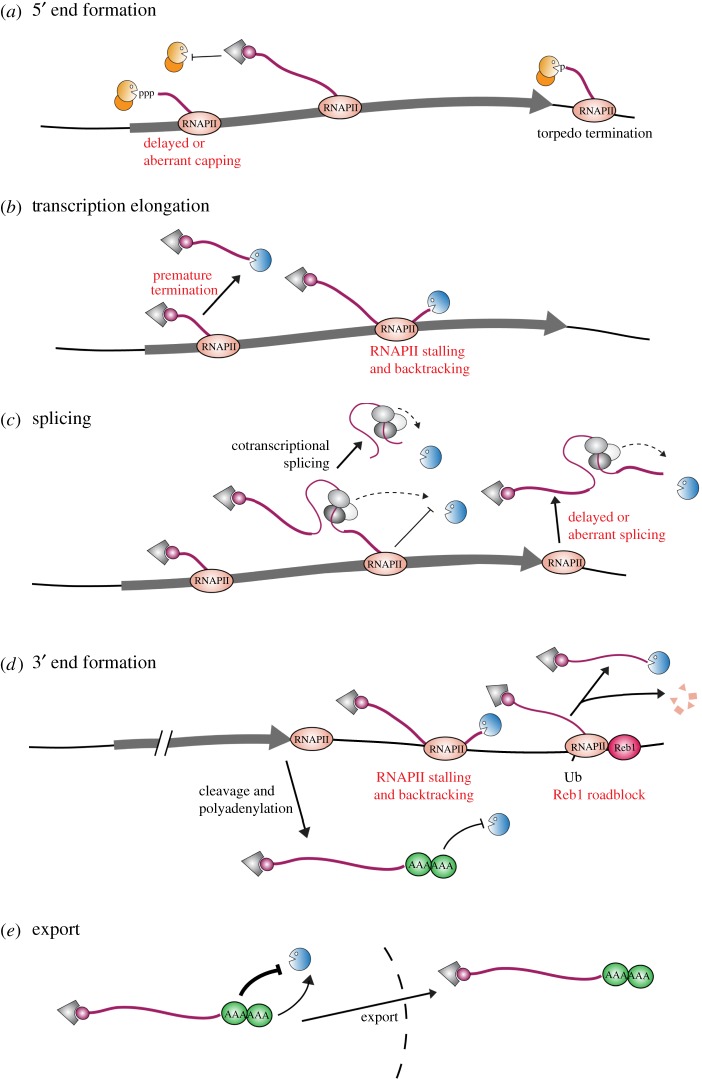
Processing and surveillance of pre-mRNAs. Multiple steps during mRNA transcription and processing are screened by surveillance activities. (*a*) Delayed or aberrant capping leads to decay by nuclear 5′ surveillance pathways. Degradation requires a pyrophosphatase activity (orange circle) to remove the triphosphate and a coupled 5′–3′ exonuclease (orange pacman). Correctly maturing transcripts are protected by the presence of the m^7^G cap and the cap binding complex (CBC; grey triangle). Following normal transcript cleavage and polyadenylation, the 3′ fragment of the nascent transcript is targeted by the 5′ exonuclease in order to terminate RNAPII transcription. (*b*) Prematurely terminated transcripts are 3′ degraded by the nuclear exosome (blue pacman). Transcription termination and surveillance can involve either complete dissociation of the polymerase (*left*) or polymerase backtracking to reveal the 3′ end, providing an entry point for the exosome (*right*). (*c*) Unspliced transcripts are targeted by the surveillance machinery. In normal mRNA biogenesis, introns are typically spliced cotranscriptionally. Excised introns must be constitutively degraded and features associated with splicing or introns may act to recruit the nuclear surveillance machinery. When introns are not efficiently removed, these factors may facilitate degradation of the entire transcript. (*d*) Aberrant 3′ end formation leads to surveillance by the nuclear exosome. In fission yeast, this can involve RNAPII stalling and backtracking downstream of the PAS (centre). Alternatively, the budding yeast protein Reb1 (red circle) can terminate transcription by functioning as a roadblock (*right*). RNAPII is ubiquitinated and degraded, and the released transcript is degraded by the nuclear exosome*.* Correctly terminated transcripts (*left*) are protected by a poly(A) tail appropriately packaged with poly(A) binding proteins (green circles). (*e*) Transcripts with prolonged nuclear retention are subject to slow, default surveillance pathways. This process appears to be facilitated in part by nuclear poly(A) binding proteins, which protect the transcript but can also stimulate decay through recruitment of the nuclear surveillance machinery.

Almost all RNA species undergo elaborate maturation processes within the nucleus. In the case of messenger RNAs (mRNAs), nascent transcripts are synthesized by RNA polymerase II (RNAPII) as pre-mRNAs consisting of both introns and exons (figure 1). Introns are removed and generally degraded, while the intervening exons are spliced together to generate the mature message. Further modifications are made to the ends of the transcript. An inverted, 7-methylguanosine cap structure is added to the 5′ end, and a polyadenylated (poly(A)) tail is synthesized at the 3′ end. In parallel, RNA binding proteins package the transcripts into export-competent mRNP particles, which are subsequently transported to the cytoplasm. These events are coordinated by the repetitive carboxy-terminal domain (CTD) of RNAPII, which acts as a general binding platform for RNA processing factors (reviewed in [1]). The heptad repeats (26 in yeast and 52 in humans) of the CTD are differentially phosphorylated throughout the transcription cycle, allowing distinct sets of maturation factors to be recruited at the correct time and place.

The complexity of nuclear RNA processing makes it inevitable that some fraction of nascent transcripts will fail to mature correctly. The accumulation of aberrant or defective transcripts represents a significant potential problem, because they could saturate the RNA processing machinery and impede the production of functional products. For example, accumulation of cryptic RNAs in yeast mutants with defective RNA degradation reduces the availability of the nuclear cap binding complex, with pleiotropic effects on gene expression [2]. Antisense transcripts can hybridize to complementary sense RNA, forming double-stranded RNAs that may enter the RNA interference pathway [3]. Excess RNA, particularly if poorly packaged, can also bind to homologous gene loci, forming harmful RNA:DNA hybrids that are associated with DNA double-strand breaks. In the cytoplasm, aberrant mRNAs may encode truncated, nonfunctional or even dominant negative proteins.

Historically, RNA quality control or ‘surveillance’ mechanisms have been difficult to examine in unperturbed systems, as defective transcripts generally constitute only a small proportion of any given endogenous mRNA species. Most studies have, therefore, relied on mutations, either in reporter transcripts or the RNA processing machinery, to artificially trigger surveillance. These analyses suggest that the surveillance machinery monitors a wide range of processing defects, including transcripts with defects in cap structure [4]; inefficient polyadenylation [5]; aberrant splicing or 3′ end formation; improper mRNP packaging [6]; or inefficient nuclear export (figure 1). These defects have little in common, but all apparently lead to recognition and destruction of the RNA by the surveillance machinery. Moreover, the same core system degrades transcripts generated by RNA polymerases I and III, which are significantly different in structure and packaging from most RNAPII products.

Surveillance pathways also degrade RNAs that result from pervasive transcription (reviewed in [7]). Eukaryotic promoters generally drive transcription initiation in both directions, but in most cases only one side results in productive gene expression [8–11]. In part, directionality is enforced through selective degradation of the upstream antisense transcript [12–16]. These RNAs are referred to as cryptic unstable transcripts (CUTs) in yeast, and promoter upstream transcripts (PROMPTs) or upstream antisense RNAs (uaRNAs) in mammalian cells. While bidirectional promoters are a prominent source of transcriptional noise, many or all active enhancer elements are also transcribed and cryptic transcription can initiate from any nucleosome free region [9]. The resulting transcripts are highly unstable in wild-type cells but accumulate when the surveillance machinery is inactive [13,16,17].

In organisms as diverse as yeast, plants and humans, RNA surveillance depends on the nuclear exosome, a complex with endonuclease and 3′ exonuclease activity. The exosome, in turn, relies on numerous cofactors to guide it to target transcripts and help initiate decay. Interestingly, many of these cofactors also function in RNA maturation, suggesting that the exosome is recruited to nascent transcripts regardless of their processing status. As discussed below, the exosome appears to act as a general scavenger of 3′ ends, potentially degrading nuclear pre-mRNAs and other transcripts by default. Correctly processed transcripts largely escape nuclear surveillance through the deposition of specific RNA binding proteins, particularly at the 3′ end, which sterically hinder exonucleolytic decay.

In this review, we discuss quality control mechanisms for RNAPII transcripts in *Saccharomyces cerevisiae* and human cells, with additional reference to RNAPI and studies in other species as appropriate. We begin with an introduction to the major cellular exonucleases and cofactors, highlighting their parallel roles in mRNA biogenesis and surveillance. Subsequently, we review mechanisms by which the exosome degrades aberrant transcripts, with a particular focus on the connections between transcription termination and surveillance.

## 2. Surveillance machinery

**Figure 2.**
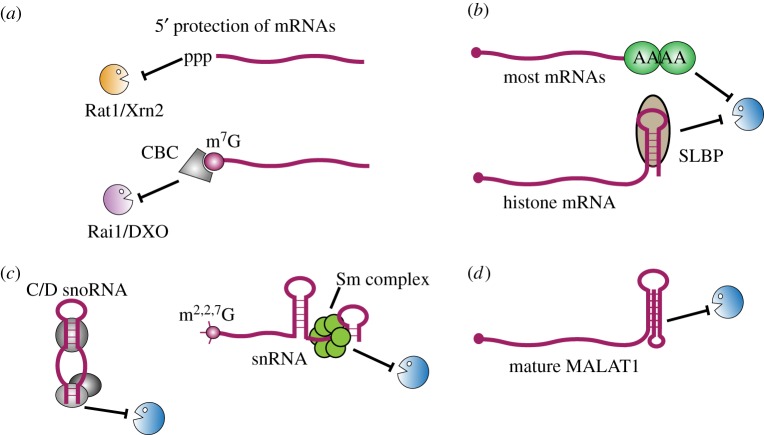
Protective features in RNA stability. (*a*) Primary RNAPII transcripts are initially protected by the terminal 5′ triphosphate, which blocks degradation by the nuclear 5′–3′ exonucleases Rat1/Xrn2. Transcripts are generally rapidly modified by addition of an inverted GpppN cap structure. This is sensitive to removal by the pyrophosphatases Rai1 and DXO, but undergoes m^7^G methylation and association with the cap binding complex (CBC), conferring pyrophosphatase resistance. (*b*) Most mRNAs are shielded at their 3′ end by a poly(A) tail packaged with poly(A) binding proteins. The non-polyadenylated, replication-dependent histone mRNAs are protected by a terminal stem–loop structure bound to the stem–loop binding protein (SLBP). (*c*) Small nucleolar RNAs (snoRNAs) and small nuclear RNAs (snRNAs) are shielded from exosome-mediated decay by specific proteins bound to the 3′ end. snRNAs and many snoRNAs are protected at their 5′ end by the trimethylated m^2,2,7^G cap. (*d*) The mature MALAT1 transcript contains a triple helix that sequesters the 3′ end and prevents 3′–5′ exonucleolytic decay.

A striking feature of eukaryotic RNA degradation and surveillance pathways is the preponderance of exonucleases, which degrade RNAs from the 5′ end (5′ exonucleases) or 3′ end (3′ exonucleases), rather than endonucleases that can cleave RNAs internally. As a consequence, accessibility of the 5′ or 3′ ends of the transcript for nuclease attack is likely to be a key feature in determining susceptibility to degradation (figure 2).

### 2.1. 5′ Exonucleases

The major 5′ exonucleases in eukaryotes are the related proteins Xrn1, which is predominantly cytoplasmic, and Rat1 (Xrn2 in humans), which is predominantly nuclear. The activities of both enzymes are largely blocked by the presence of a 5′ triphosphate [18], which is initially present on all newly synthesized transcripts. This protection can be seen during degradation of the excised 5′ external transcribed sequence (5′ETS) spacer of the pre-ribosomal RNA (pre-rRNA). The 5′ region, which carries the 5′ triphosphate, is degraded by the exosome (see below), whereas the 3′ region, which is generated by cleavage and carries a 5′ monophosphate, is degraded by Rat1 [19,20]. Similarly, 3′ cleavage of the pre-rRNA allows entry of Rat1, which degrades the downstream nascent transcripts. The pre-mRNA 3′ cleavage and polyadenylation machinery also leaves a 5′ monophosphate, allowing Rat1/Xrn2 to degrade the downstream nascent transcript [19–21]. Transcription is then terminated by Rat1/Xrn2, presumably acting when it catches the transcribing polymerase, in a phenomenon referred to as ‘torpedo’ termination [19–21].

Nascent RNAPII transcripts are further protected by addition of a 7-methylguanosine (m^7^G) cap to the 5′ triphosphate end of the RNA. This reaction occurs shortly after transcription initiation, usually within the first 50 nt. Capping defects can be induced by inactivation of the capping machinery itself, or indirectly through mutations in RNAPII [22]. Rat1 forms a complex with the pyrophosphatase Rai1 and transcripts that fail to be capped, or on which the cap is not 7-methylated, are rapidly decapped and dephosphorylated by Rai1 [23]. This exposes the transcript to degradation by Rat1 [19,22,24–26]. Capped transcripts are further protected by the nuclear cap binding complex (CBC), comprising Cbc1–Cbc2 in yeast and CBP80–CBP20 in humans. CBC directly blocks access to the 5′ cap by the decapping enzyme Dxo1, which is homologous to Rai1 and can also initiate degradation [27,28].

Since decapping leaves a 5′ monophosphate, it seems likely that RNAPII will also be subject to torpedo termination by Rat1 when capping is defective. Notably, the sensitivity of degradation systems to 5′ nucleotide status is conserved in evolution, because the major bacterial endonuclease RNase E is active only on substrates with a 5′ monophosphate [29].

### 2.2. The exosome

In eukaryotes, the major 3′ exonuclease activity during RNA surveillance is supplied by the nuclear exosome complex. The exosome was originally defined for its role in the processing of precursors to stable RNAs; rRNA, small nucleolar RNA (snoRNA) and small nuclear RNA (snRNA) are all processed at least in part by the exosome [30,31]. Subsequently, the exosome was shown to target a wide variety of transcripts, including defective pre-rRNAs, pre-tRNAs, aberrant mRNAs and transcripts arising from pervasive transcription [13,16,17,32–36]. Exosome structure and function is the subject of several excellent reviews [37–39], and will be discussed only briefly here.

The exosome consists of a central hexameric ring comprising six proteins, with an additional three subunits layered on top. These nine proteins are arranged in a barrel surrounding a central channel just wide enough to accommodate single-stranded RNA. Collectively, this complex is referred to as the exosome core, and is structurally conserved in Archaea. In eukaryotes, catalytic activity is supplied by the associated, highly processive 3′ to 5′ exonuclease Dis3/Rrp44. Dis3 is positioned at the base of the barrel, and approximately 30 nt of single-stranded RNA must be threaded through the central channel to reach the active site [40,41]. Dis3 has an additional PIN endonuclease domain, but the range of *in vivo* targets for this endonuclease activity is unclear; the only confirmed targets are the 5′ETS and pre-5.8S regions of the pre-rRNA. The nuclear exosome can also associate with the distributive 3′ to 5′ exonuclease Rrp6, assisted by its cofactor Rrp47 (C1D in human cells) [42–44]. Dis3 and Rrp6 show some functional specialization, but there is considerable overlap in their target sets [32,33].

### 2.3. Nuclear exosome cofactors; TRAMP, NNS, NEXT

The eukaryotic exosome core is relatively inactive, making its function heavily reliant on cofactors. A well-studied cofactor is the Trf4/5–Air1/2–Mtr4 polyadenylation (TRAMP) complex. In *S. cerevisiae,* TRAMP consists of a poly(A) polymerase (PAP) (either Trf4 or Trf5), a zinc-finger, RNA binding protein (Air1 or Air2) and the RNA helicase Mtr4 [16,45,46]. Trf4/5 adds a short oligo(A) tail to the transcript end, which Mtr4 threads into the exosome central channel. Air1 and Air2 are thought to aid in substrate binding. *In vivo* cross-linking experiments with Dis3 reveal a high fraction of reads with non-templated A-tails, suggesting that TRAMP-mediated polyadenylation generally precedes exosome-mediated decay [33]. Indeed, TRAMP plays an essential role in the degradation of nearly all surveillance targets of the exosome in yeast, including defective, hypomodified pre-tRNA_i_^Met^ [47], defective pre-rRNAs [48] and cryptic RNAPII transcripts [16].

The role of Mtr4 and the exosome in ribosome production is complex. Mtr4 acts in ‘constitutive’ pre-rRNA processing steps: 3′ trimming of the pre-5.8S rRNA and turnover of the excised 5′ETS spacer. Specific adaptor proteins, the ribosome biogenesis factors Nop53 and Utp18, interact with the arch domain of Mtr4 during recruitment to the pre-5.8S rRNA and 5′ETS, respectively [49]. Notably, both the arch domain of Mtr4 and the arch-interacting motif of Nop53 are conserved in higher eukaryotes. When ribosome synthesis is proceeding normally, exosome activity is terminated at the 5′ end of the pre-rRNA or close to the mature 3′ end of the 5.8S rRNA. However, on pre-rRNAs that fail to undergo cotranscriptional cleavage, recruitment of the exosome leads to degradation of the entire pre-rRNA.

There is little evidence that the pre-rRNA processing steps involve the TRAMP complexes, which probably function only in pre-rRNA surveillance. Consistent with this, loss of Trf5 was shown to partially rescue ribosome synthesis in several different pre-ribosome assembly mutants [50,51]. Moreover, hyperadenylated pre-rRNAs accumulate on depletion of Mtr4 [52], showing that the Trf–Air module of TRAMP can be recruited independently of Mtr4 and, presumably, of the exosome [51,52].

Notably, the processing roles of Mtr4 and exosome take place in the context of a specific, on-pathway pre-ribosomal particle, in which the interactions have presumably been fine-tuned through evolution. During surveillance, in contrast, TRAMP, Mtr4 and the exosome must respond to a multitude of different, off-pathway particles with diverse defects in RNA processing, RNA folding and RNA–protein interactions. Similar issues must arise during surveillance of other RNA classes; the list of defects that could potentially arise seems almost endless. We therefore predict that the surveillance system is keyed to recognize ‘generic’ attributes, and uses general adaptor proteins, rather than recruitment by specific features of any particular, misassembled RNA–protein complex.

**Figure 3.**
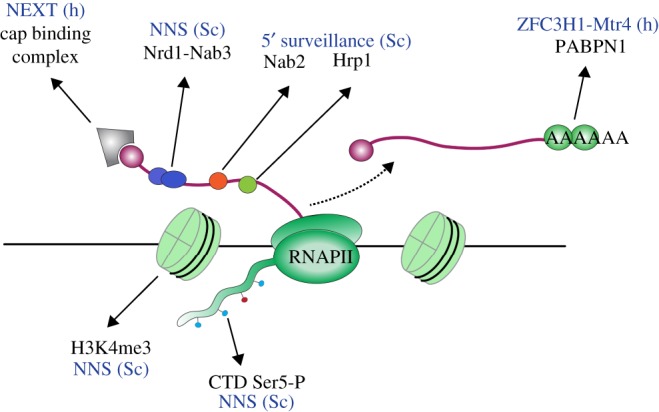
Pro-surveillance factors in RNAPII transcription units. Nascent transcripts contain numerous features which facilitate the recruitment of surveillance factors. Importantly, these factors are also present during normal RNA biogenesis, which presumably allows the nuclear surveillance machinery to ‘inspect’ all nascent RNAs. Details are discussed in the text. h, human; Sc, *Saccharomyces cerevisiae*.

TRAMP-mediated surveillance of RNAPII transcripts is usually coupled to transcription termination by the Nrd1–Nab3–Sen1 (NNS) complex [14,15,53]. Nrd1 and Nab3 are RNA binding proteins that each bind a short consensus motif [54,55]. However, these sequence elements are abundant throughout the genome and usually insufficient to drive binding [56]. Nrd1–Nab3 recruitment is aided by a physical interaction between Nrd1 and phosphorylated serine 5 (Ser5-P) within the CTD of RNAPII [57,58] (figure 3). Ser5-P is ubiquitously present during early transcription and, probably as a result, Nrd1 and Nab3 are broadly recruited to the 5′ ends of nascent transcripts [54–56,59]. Once bound, Nrd1 and Nab3 can engage Sen1, an ATP-dependent, 5′–3′ RNA and DNA helicase. Sen1 is the putative transcription termination factor, which may displace polymerase and free the nascent transcript for degradation by the TRAMP–exosome complex [14,15,53,60–62]. Termination by NNS is further promoted by interactions with histone H3 modified by lysine 4 trimethylation (H3K4me3) [63], a hallmark of the 5′ regions of protein-coding genes.

The NNS pathway also participates in the maturation of snoRNAs [64], promoting 3′ processing rather than complete degradation. This is possible because specific snoRNA-binding proteins mark the mature 3′ end and block further degradation by the exosome.

The NNS pathway seems to be unique to budding yeasts; there are apparent human homologues of Nrd1 (SCAF8/RBM16) and Sen1 (Senataxin), but these are not known to form a complex with a Nab3 homologue. By contrast, the TRAMP complex is well conserved [65], but human TRAMP is confined to the nucleolus, where it presumably participates in pre-rRNA surveillance [66]. hMtr4 is distributed more broadly throughout the nucleus, and can also associate with the RNA binding proteins RBM7 and ZCCHC8 to form the nuclear exosome targeting (NEXT) complex [66]. Both RBM7 and ZCCHC8 are restricted to the nucleoplasm, and NEXT participates in surveillance of RNAPII transcripts, including the degradation of cryptic transcripts. Additionally, NEXT facilitates the termination and 3′ end processing of human snRNAs [66,67]. NEXT is recruited to target transcripts through interactions with the nuclear CBC [68] and, therefore, binds promiscuously during early RNAPII transcription (figure 3). Subsequently, the CBC–NEXT complex triggers early termination and degradation of target transcripts [69]. These features are analogous to the NNS pathway, despite the lack of sequence homology between the individual components.

## 3. Surveillance throughout the transcription cycle

### 3.1. Transcription elongation

During transcription elongation, the 3′ end of the nascent transcript is buried within the active site of RNAPII and inaccessible to 3′ exonucleases. It seems likely that exosome-mediated surveillance will first require transcription termination and dissociation of the polymerase. Indeed, this appears to be the mechanistic basis for the NNS and NEXT pathways. There is, however, another potential pathway that might allow the exosome access to the 3′ ends of nascent transcripts. When transcription elongation is impeded, the polymerase can backtrack—sliding backwards along the DNA and extruding the 3′ end of the nascent transcript from the catalytic site (reviewed in [70]). Backtracked RNAPII is generally rescued by transcription factor IIS (TFIIS; Dst1 in yeast) [71], which activates the intrinsic hydrolytic activity of RNAPII. This cleaves the nascent transcript and realigns the 3′ end within the active site, allowing transcription to resume.

In principle, extended backtracking might also lead to the extruded 3′ end projecting from the polymerase to a sufficient extent to allow binding of the surveillance machinery. This could provide an entry point for the nuclear exosome to degrade the nascent transcript, displacing RNAPII in the process [72]. Consistent with this model, depletion of Dis3 in the fission yeast *Schizosaccharomyces pombe* results in extended transcripts with elevated RNAPII occupancy downstream of the poly(A) site [72]. RNAPII is known to pause downstream of the poly(A) site, allowing time for cotranscriptional cleavage and polyadenylation [73,74]. This pause is proposed to favour backtracking, leading to transcription termination via the exosome [72]. For transcripts that are rapidly cleaved and polyadenylated prior to transcription termination, the exosome would degrade only the sequence downstream of the poly(A) site. The remainder of the transcript would be protected due to its separation from the elongation complex by 3′ cleavage. Were RNA cleavage delayed, the exosome could destroy the entire transcript, providing quality control for timely 3′ end formation. RNAs lacking bona fide polyadenylation signals or transcripts which fail to correctly assemble the cleavage and polyadenylation complex would then be degraded by default (figure 1). This model might also rationalize observations from *S. cerevisiae* which implicate the exosome in mRNA transcription termination [32,75]. Backtracking has also been invoked to explain transcription termination in human cells [76].

### 3.2. Splicing

Unspliced pre-mRNAs are very rapidly degraded when splicing is delayed, indicating active surveillance. However, the mechanism and factors involved remain obscure. In yeast and humans, splicing generally occurs cotranscriptionally, so introns and splicing factors are usually removed before the 3′ end is exposed by transcription termination. Since excised introns must always be degraded, or trimmed to release internal snoRNAs, it is possible that features or factors associated with splicing would act to specifically recruit the RNA degradation system. When splicing is delayed, the 3′ end is no longer protected by the transcription elongation complex and these factors could facilitate degradation of the entire transcript. Degradation could be induced through direct recruitment of the exosome by one or more splicing factors, or through specific decay-promoting sequences within introns. The clearest example is the binding of Mmi1 to fission yeast introns [77]. Mmi1 recruits the exosome to a subset of unspliced introns, facilitating degradation of the entire RNA. However, these transcripts will generally be terminated by the canonical cleavage machinery, so degradation either outcompetes polyadenylation or is able to overcome the protection a poly(A) tail would normally confer. Whether additional intron-associated factors participate in surveillance is unclear, so this model remains somewhat speculative. However, if multiple exosome recruitment pathways exist, loss of a single factor might not have dramatic effects.

### 3.3. Termination

Eukaryotic pre-mRNAs are generally terminated following cotranscriptional endonuclease cleavage downstream of the open reading frame. In nearly all instances, pre-mRNA cleavage is coupled to the addition of a poly(A) tail. In contrast to alternative termination mechanisms, pre-mRNA cleavage and polyadenylation is not clearly associated with exosome-mediated processing or decay. This is usually attributed to the protective effects of poly(A) binding proteins (poly(A) BPs), which coat the nascent poly(A) tail during its synthesis and are thought to fend off exonuclease attack [78]. This hypothesis has been most directly tested in *S. cerevisiae*, where rapid depletion of the nuclear poly(A) BP Nab2 dramatically destabilizes newly synthesized, polyadenylated mRNAs [79]. This phenotype is alleviated when the exosome is also inactivated, indicating that Nab2 protects poly(A) tailed transcripts from the nuclear exosome (figure 1). Whether this role for nuclear poly(A) BPs is conserved in higher eukaryotes remains uncertain. Human cells encode multiple, potentially redundant, nuclear poly(A) BPs and their roles in mRNA stability remain to be clarified.

In contrast to the protective effects of canonical cleavage and polyadenylation, transcripts terminated by alternative mechanisms are generally highly unstable. The clearest example is NNS-mediated termination, which usually facilitates termination early in the transcription cycle. However, even full-length transcripts are destabilized if termination is not coupled to polyadenylation and poly(A) BP binding. In *S. cerevisiae*, binding sites for the DNA-binding protein Reb1 are enriched in intergenic regions downstream of poly(A) sites [80]. DNA-bound Reb1 acts as an orientation-sensitive roadblock for RNAPII, which stalls and is ultimately ubiquitinated to induce transcription termination [80]. The nascent RNA is released with a 3′ monophosphate, and rapidly degraded by the TRAMP–exosome complex. In alternative pathways, readthrough pre-mRNA transcripts can be terminated by the NNS complex or following transcript cleavage in double-stranded regions by the RNase III homologue Rnt1 [81,82]. In each case, the terminated transcripts lack a poly(A) tail and are rapidly degraded by the nuclear exosome.

Underlining the importance of 3′ protection, stable but non-polyadenylated RNAPII transcripts all carry specialized 3′ structures and/or specific RNA 3′-end binding proteins (figure 2). The major form of the highly expressed human long noncoding RNA (lncRNA) MALAT1 has a 3′ terminus that is generated by RNase P cleavage at a tRNA-like element [83]. The resulting transcript ends in an A-rich stretch that interacts with two upstream U-rich elements to form a triple-helix structure, which sequesters the 3′ end of the transcript from exonucleases [84,85]. Interestingly, triple-helix formation can also be used to protect a canonical poly(A) tail. For example, the viral noncoding and nuclear-retained RNA PAN carries internal U-rich elements that sequester the poly(A) tail within a triple helix [86] and protect it from exosome-mediated decay [87,88]. Similar structures have been identified in other transcripts, suggesting triple helices could be a widespread mechanism to protect the poly(A) tails of nuclear-retained transcripts [89,90].

In metazoans, the majority of histone proteins are encoded by replication-dependent histone genes (reviewed in [91]). These histone mRNAs are synchronously up-regulated during S phase to support DNA replication. The transcript 3′ end is formed through endonucleolytic cleavage of the pre-mRNA by CPSF73, guided by the U7 snRNA. CPSF73 is also required for canonical cleavage and polyadenylation, but these histone mRNAs are not polyadenylated. Instead, a highly conserved, terminal stem–loop sequence is specifically bound by the stem–loop binding protein (SLBP), which confers protection against 3′ degradation [83].

In yeast, 3′ processing of most snRNAs or snoRNAs is initiated by cotranscriptional cleavage by the endonuclease Rnt1, a homologue of RNase III, which cleaves both sides of a stem–loop structure. The cleaved pre-sn(o)RNA is probably initially protected by 3′ binding of the La protein homologue Lhp1 and/or the Lsm2-8 complex to oligo(U) tracts [92–95], allowing time for assembly of the sn(o)RNP complexes. Loss of the proteins that bind the snRNA or snoRNA 3′ terminus leads to complete degradation of the mature sn(o)RNA region [95,96] (figure 2).

### 3.4. mRNP packaging

In principle, poorly packaged transcripts which remain bound to chromatin could base pair with the complementary DNA strand. For example, exosome inactivation in human cells leads to the accumulation of enhancer-associated ncRNAs (eRNAs), which generate RNA:DNA hybrids (R-loops) [97]. In both yeast and humans, R-loops are associated with genome instability but are efficiently removed by the RNase H endonuclease [98–100]. RNase H selectively cleaves RNA that is base-paired with DNA, and presumably provides an entry point for 5′ and 3′ exonucleases to degrade the entire RNA molecule. Thus, RNase H may function as an additional layer of RNA surveillance to ensure transcript quality.

## 4. Dual roles of nuclear poly(A) binding proteins in stability and decay

Despite its role in mRNA stability (see above), Nab2 has also been implicated in RNA surveillance pathways. Nab2 directs the degradation of pre-mRNAs by Rrp6 [101,102] and autoregulates the *NAB2* mRNA by recruiting the TRAMP–exosome complex [103,104]. Nab2 was also proposed to target CUTs, which generally lack polyadenylation signals and are not polyadenylated [105]. Notably, while Nab2 is enriched at the poly(A) tail, it binds promiscuously throughout transcripts at non-poly(A) sites [105]. These observations indicate that Nab2 can stimulate degradation when not associated with the poly(A) tail.

The human nuclear protein PABPN1 is not related to Nab2 in sequence, but plays an analogous role in nuclear surveillance, targeting a wide range of nuclear substrates. These include spliced genes that encode snoRNAs but no protein product, cryptic transcripts arising from divergent promoters, intronless transcripts and partially spliced mRNAs [88,106–109]. In contrast to Nab2, PABPN1 targets full-length and polyadenylated transcripts, suggesting that PABPN1 stimulates decay while bound to the poly(A) tail. This activity may be enabled by PABPN1's additional role in polyadenylation (reviewed in [78]). On long poly(A) tails, PABPN1 facilitates the distributive extension of the tail by PAP [110], perhaps creating a free 3′ end accessible to the exosome. Consistent with this notion, PAP activity is apparently required for efficient degradation [109].

In addition, PABPN1 has been reported to recruit the zinc-finger protein ZFC3H1, which acts together with Mtr4 to stimulate exosome degradation of the transcript [108,111]. This surveillance pathway is apparently absent from budding yeast but conserved in fission yeast. Red1 is the homologue of ZFC3H1 and works with Mtr4-like 1 (Mtl1) to process snoRNAs and degrade CUTs, some pre-mRNAs, and meiotic mRNAs, which must be eliminated in mitotic cells [112]. The Mtl1–Red1 core (MTREC) complex is directed to target transcripts by a variety of adaptors, including the PABPN1 homologue Pab2 and the PAP Pla1 [112–117]. The MTREC complex also associates with another 3′ end formation factor Hrp1/Nab4 [117]. In budding yeast Hrp1 is required for pre-mRNA cleavage, binding an upstream UAUAUA sequence [118]. However, like Nab2, Hrp1 also binds at alternative sites and contributes to ncRNA degradation [105] (figure 3).

The dual nature of nuclear poly(A) binding proteins in RNA stability and decay may reflect distinct roles during mRNA biogenesis. Following transcription through the cleavage site, mRNAs are rapidly polyadenylated, a process which is coupled to packaging by poly(A) BPs. Delayed poly(A) binding will expose the 3′ end to exosome-mediated degradation before the transcript can be exported to the cytoplasm. However, because nuclear poly(A) BPs also recruit the exosome, any transcript with significantly delayed nuclear export will eventually be degraded by default, albeit with much slower kinetics (figure 1). In this respect, it is notable that errors in splicing frequently prevent export, and that Nab2, Pab2 and PABPN1 each target intron-containing mRNAs [101,109,112,113]. In human cells, sensitivity to PABPN1-mediated decay is indeed correlated with prolonged nuclear retention [109,119].

## 5. Controlling pervasive transcription

**Figure 4.**
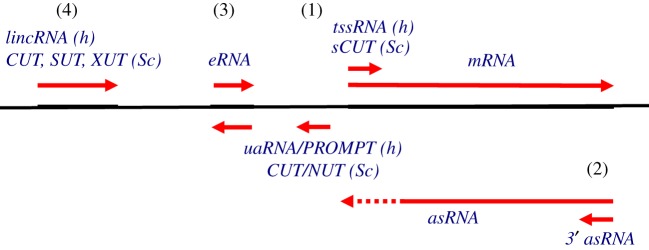
Major classes of noncoding RNA. The regions surrounding eukaryotic protein-coding genes generate a set of ncRNAs in addition to the mRNA transcript. These include: (1) short divergent promoter associated transcripts from the nucleosome-free promoter region, (2) antisense (as) transcripts from the nucleosome-depleted terminator region, (3) enhancer-associated eRNAs. In addition, (4) a range of ncRNAs are transcribed from intergenic locations. h, human; Sc, *Saccharomyces cerevisiae*.

Arguably the most significant role of the nuclear surveillance machinery is the removal of huge numbers of different RNAs that arise from pervasive transcription. This term was initially coined in response to the unexpected finding that a large fraction of the human genome is transcribed into unstable RNAs with little or no protein-coding potential [120]. Subsequently, several different classes of ncRNA have been defined and included under this general heading (figure 4). In general, noncoding regions diverge rapidly during evolution, presenting clear problems for their efficient recognition by highly conserved, protein-based surveillance systems. As we discuss below, the surveillance machinery seems to recognize common features of ncRNA biogenesis rather than sequence.

Genome-wide studies indicate that eukaryotic promoters are intrinsically bidirectional [8–11,121]. At many promoters, polymerase initiates transcription equally in both directions, but transcripts in the ‘upstream’ direction are quickly eliminated by the nuclear surveillance machinery. In both yeast and humans, this surveillance is intimately linked to the mechanism of premature transcription termination.

In *S. cerevisiae*, promoter-associated ncRNA transcription is terminated through the NNS pathway, and the resulting transcripts are degraded by the TRAMP–exosome complex. Nrd1 and Nab3 binding motifs are enriched in transcripts generated upstream of bidirectional promoters, also termed NUTs, and depleted within protein-coding transcripts [53]. This asymmetry presumably favours NNS-dependent termination in upstream regions, but whether it is sufficient to explain the difference in stability between CUTs/NUTs and mRNAs is less clear. Nrd1 and Nab3 binding elements are of low complexity and generally abundant even in protein-coding genes [54–56]. Moreover, cross-linking experiments suggest Nrd1 and Nab3 bind promiscuously to most RNAPII transcripts [53–56].

An additional determinant may be the phosphorylation status of RNAPII. Early transcription is characterized by high Ser5 and low Tyr1, Ser2 and Ser7 phosphorylation levels along the CTD [122,123]. This combination of marks, termed ‘initiation state’, helps recruit the NNS complex to nascent transcripts [57,58,122]. For most genes, RNAPII remains in initiation state through the first approximately 150 nt of RNA, roughly corresponding to the first nucleosome [123]. Within this window, polymerase is particularly prone to premature termination and surveillance [105,123]. As transcription continues, the CTD is gradually reconfigured to an elongation state [123]. Most notably, Ser5 phosphorylation is reduced, rendering the transcript less prone to NNS recruitment and transcription termination. Simultaneously, an increase in Ser2 phosphorylation facilitates the recruitment of canonical cleavage and polyadenylation factors [124].

Remarkably, this transition apparently fails to take place during CUT transcription, with polymerase instead remaining stuck in initiation state [123], providing an extended window for NNS-dependent termination. Moreover, the failure to transition to elongation state prevents the recruitment of factors that might otherwise facilitate termination at cryptic poly(A) signals and yield a stable transcript. These factors imply that CUTs are in some sense ‘fated’ for degradation by the surveillance machinery.

Analogous mechanisms control divergent transcription in humans. Regions upstream of bidirectional promoters are enriched for consensus poly(A) signal (PAS) motifs, aiding early termination by the conventional cleavage and polyadenylation machinery [12,125]. These prematurely terminated transcripts are rapidly degraded by the nuclear exosome, typically in concert with the NEXT complex [66,68]. When present in the sense direction, PAS motifs are usually suppressed by the presence of U1 snRNP along the RNA [125,126]. Together, these features constitute the ‘U1–PAS axis’ [125], and help enforce transcription directionality. However, even sense transcripts are sometimes subject to premature cleavage and degradation [127].

At first glance, it is surprising that conventional termination should be coupled to decay. However, despite being processed by the canonical cleavage and polyadenylation machinery, uaRNAs are often not polyadenylated [108]. This suggests divergent transcripts are targeted soon after transcript cleavage, perhaps during an early, distributive phase of polyadenylation when the transcript is still unprotected by nuclear poly(A) BPs. This raises the obvious question of why mRNAs are not also susceptible to NEXT-mediated decay. The answer has been proposed to relate to differences in transcript length between uaRNAs and mRNAs [108]. NEXT is recruited by the CBC and, like NNS, preferentially localizes near the 5′ ends of nascent RNAs [68,69]. Cleavage in this region could favour decay triggered by NEXT and thus disfavour polyadenylation. Consistent with this hypothesis, NEXT is most active on transcripts shorter than 2 kb [108]. When transcript cleavage happens far downstream, as is usually the case for mRNAs, polyadenylation should be favoured.

As with yeast, it is unlikely that any one characteristic is sufficient for the mammalian surveillance machinery to distinguish mRNAs from transcriptional noise. Another important feature may be splicing, which has long been associated with RNA stability and surveillance. In mammalian cells, intronless transcripts tend to be weakly expressed and unstable [119] and, while nearly all mammalian mRNAs are spliced, the vast majority of divergent transcripts are not. The connection between a lack of splicing and uaRNA instability may be direct, because splicing deposits the mRNA export factor REF on nascent transcripts, where it physically interacts with CBC [128]. Notably, REF directly competes with Mtr4 for access to CBC [129], indicating that the export machinery antagonizes nuclear surveillance. Moreover, REF overexpression is sufficient to protect some divergent transcripts from exosome-mediated decay [129]. Somewhat surprisingly, these effects are independent of the NEXT complex, suggesting Mtr4 can also act independently or with other exosome adaptors to degrade divergent transcripts. A plausible candidate is the PABPN1–PAP–ZFC3H1 pathway, which helps to degrade a subset of uaRNAs [107–109].

## 6. Slow, default decay for nuclear-retained transcripts?

In addition to CUTs, yeast also produce stable unannotated transcripts (SUTs). Whereas CUTs are only observable when the surveillance machinery is compromised, SUTs are readily detectable in wild-type cells [9]. The greater stability of SUTs might be explained by ‘mRNA-like’ processing [105], because they undergo 3′ cleavage and polyadenylation, with recruitment of Nab2 and/or Pab1 to the poly(A) tail. However, unlike most mRNAs, SUTs are not extensively bound by the export factor Mex67 or the cytoplasmic localization protein Hek2 [105]. These observations suggest SUTs are predominantly restricted to the nucleus, and perhaps targeted nonspecifically by nuclear RNA decay pathways. Consistently, SUTs are stabilized in strains lacking a functional nuclear exosome [32,130]. A subset of SUT-like RNAs are exported and strongly stabilized in the absence of the cytoplasmic exonuclease Xrn1, resulting in the designation of Xrn1-sensitive unstable transcripts (XUTs) [130].

SUT biogenesis appears homologous to nuclear lncRNAs in higher eukaryotes. Mammalian lncRNAs bear a striking resemblance to mRNAs; both classes are capped and polyadenylated, sometimes spliced, and similar in length [131]. However, lncRNAs usually lack the strong export and translation signals typical of most mRNAs, and are more frequently confined to the nucleus [132]. Only a small proportion of lncRNAs have defined functional roles, and few show any meaningful evolutionary conservation [119].

Like SUTs, nuclear lncRNAs are significantly less stable than their cytoplasmic mRNA counterparts [119]. The relative instability of SUTs and nuclear lncRNAs is commonly attributed to their inefficient processing and export, rather than specific recognition by quality control pathways. In general, species that take longer to exit the nucleus should be more susceptible to nuclear degradation. This ‘kinetic competition’ model has also been invoked to explain mRNA surveillance. In both yeast and humans, mistakes in splicing, 3′ end formation or mRNP packaging can inhibit nuclear export. In this way, some aberrant mRNAs may be degraded by default on account of their prolonged nuclear retention (figure 1).

## 7. Conclusion and perspectives

RNAs carry out diverse functions within cells, providing both the machinery for protein production and the information to programme its activity. In order to function in these distinct activities, all RNAs undergo elaborate maturation processes within the nucleus. During these complex processing and assembly pathways, defects inevitably arise in some RNA transcripts and RNA–protein complexes, which must be systematically removed by quality control pathways. In this review, we proposed that the identification and degradation of defective RNAs, and enormous numbers of spurious transcripts, does not require recognition of specific ‘bad’ features. Rather the surveillance system will, by default, target almost all RNAs. Transcripts that undergo correct and timely maturation acquire protective features that help them evade the activities of the surveillance system. This model potentially explains how eukaryotes can tolerate the synthesis of huge numbers of diverse ncRNA transcripts, which are constantly cleared by rapid degradation. Moreover, it has been proposed, on thermodynamic grounds, that pervasive transcription is an inevitable feature of eukaryotic genomes [133]. The development of a surveillance ready transcription system may, therefore, have been a prerequisite for the evolution of the very large genomes found in many higher eukaryotes.

Although our understanding of these surveillance pathways is rapidly expanding, many questions remain unanswered. In particular, a quantitative description of RNA surveillance is needed. Discrimination between normal and defective transcripts must require an input of energy, in order to avoid violating the second law of thermodynamics. This energy input could be supplied by ATP-dependent RNA helicases, potentially explaining why helicases such as Mtr4 are critical components of essentially all surveillance pathways. However, a mechanistic understanding of the connection between ATP expenditure and RNA surveillance remains elusive. An additional avenue for future research is the role of RNA modifications such as m^6^A and m^1^A, which in recent years have been implicated in multiple aspects of the mRNA life cycle [134]. Nascent mRNAs must presumably be screened for the ‘correct’ combination of methylation marks, but how this is achieved is still unclear. In the future, the application of new techniques such as CRISPR, cross-linking-immunoprecipitation and single-RNA fluorescence microscopy will enable these questions and others to be addressed.

## Data accessibility

This article has no additional data.
